# Molecular analysis of volatile metabolites released specifically by *staphylococcus aureus* and *pseudomonas aeruginosa*

**DOI:** 10.1186/1471-2180-12-113

**Published:** 2012-06-20

**Authors:** Wojciech Filipiak, Andreas Sponring, Maria Magdalena Baur, Anna Filipiak, Clemens Ager, Helmut Wiesenhofer, Markus Nagl, Jakob Troppmair, Anton Amann

**Affiliations:** 1 Breath Research Institute of the Austrian Academy of Sciences, Rathausplatz 4, A-6850, Dornbirn, Austria; 2 Univ. Clinic for Anesthesia, Innsbruck Medical University, Anichstr. 35, Innsbruck, A-6020, Austria; 3 Department of Hygiene, Microbiology and Social Medicine, Division of Hygiene and Medical Microbiology, Innsbruck Medical University, Fritz-Preglstraße 3, Innsbruck, A-6020, Austria; 4 Daniel-Swarovski Research Laboratory, Department of Visceral-, Transplant- and Thoracic Surgery, Innsbruck Medical University, Innrain 66, A-6020, Innsbruck, Austria

**Keywords:** Volatile organic compounds (VOCs), Gas chromatography mass spectrometry (GCMS), Breath analysis, *In vitro* headspace sampling, Adsorptive enrichment, Multibed sorption tubes, Volatile metabolites, *Staphylococcus aureus*, *Pseudomonas aeruginosa*

## Abstract

**Background:**

The routinely used microbiological diagnosis of ventilator associated pneumonia (VAP) is time consuming and often requires invasive methods for collection of human specimens (e.g. bronchoscopy). Therefore, it is of utmost interest to develop a non-invasive method for the early detection of bacterial infection in ventilated patients, preferably allowing the identification of the specific pathogens. The present work is an attempt to identify pathogen-derived volatile biomarkers in breath that can be used for early and non- invasive diagnosis of ventilator associated pneumonia (VAP). For this purpose, *in vitro* experiments with bacteria most frequently found in VAP patients, i.e. *Staphylococcus aureus* and *Pseudomonas aeruginosa*, were performed to investigate the release or consumption of volatile organic compounds (VOCs).

**Results:**

Headspace samples were collected and preconcentrated on multibed sorption tubes at different time points and subsequently analyzed with gas chromatography mass spectrometry (GC-MS). As many as 32 and 37 volatile metabolites were released by *S. aureus* and *P. aeruginosa*, respectively. Distinct differences in the bacteria-specific VOC profiles were found, especially with regard to aldehydes (e.g. acetaldehyde, 3-methylbutanal), which were taken up only by *P. aeruginosa* but released by *S. aureus*. Differences in concentration profiles were also found for acids (e.g. isovaleric acid), ketones (e.g. acetoin, 2-nonanone), hydrocarbons (e.g. 2-butene, 1,10-undecadiene), alcohols (e.g. 2-methyl-1-propanol, 2-butanol), esters (e.g. ethyl formate, methyl 2-methylbutyrate), volatile sulfur compounds (VSCs, e.g. dimethylsulfide) and volatile nitrogen compounds (VNCs, e.g. 3-methylpyrrole).

Importantly, a significant VOC release was found already 1.5 hours after culture start, corresponding to cell numbers of ~8*10^6^ [CFUs/ml].

**Conclusions:**

The results obtained provide strong evidence that the detection and perhaps even identification of bacteria could be achieved by determination of characteristic volatile metabolites, supporting the clinical use of breath-gas analysis as non-invasive method for early detection of bacterial lung infections.

## Background

Pneumonia is the most common cause of death related to infectious diseases. Even after aggressive antimicrobial treatment pneumococcal pneumonia causes mortalities of up to 10% [1]. Children and young adults are susceptible to lower respiratory tract infection typically caused by *Staphylococcus aureusHaemophilus influenzae* and *Pseudomonas aeruginosa*[2]. Moreover, *P. aeruginosa* colonization of the lung is frequently found in cystic fibrosis patients, which worsens the prognosis of this disease [3]. Pneumonia signifcantly increases the average duration of intensive care unit (ICU) stays and mortality [4]. The diagnosis of nosocomial pneumonia often requires invasive and time consuming methods (e.g. bronchoscopy) [5]. Therefore, it is of utmost interest to develop a non-invasive method for the early diagnosis of this disease, preferably allowing the identification of the specific pathogens.

Attempts on screening of volatile bacterial metabolites for detection and classification of virulent bacteria was already undertaken in the past. However, the vast majority of studies on volatile organic compounds (VOCs) released from bacteria included qualitative analyses only [6-10]. Also direct mass spectrometric methods were used for the investigation of VOC release, comprising selected ion flow tube mass spectrometry (SIFT-MS) [11,12] and proton transfer reaction mass spectrometry (PTR-MS) [13-15]. However, especially in the case of PTR-MS the identification of VOCs is questionable, since several compounds may undergo fragmentation and contribute to one *m/z* signal [16,17]. Nevertheless, encouraging results were already reported, showing that some microorganisms have their own expression patterns of enzymes, producing a characteristic range of volatile metabolites [18-21]. This prompted us to further develop exhaled breath analysis for the diagnosis of bacterial lung infections [22,23]. Although changes in the VOC patterns in breath have not been extensively investigated so far, detection of bacterial infections by exhaled air analysis has been proposed by sensor technology [24,25]. This approach is still in its infancy and often under critical review due to the use of complex mathematical pattern recognition instead of clear identification of specific VOCs. Sensor systems such as the electronic nose systems (EN) are often unspecific and do generate a pattern of responses for the sensors. Neuronal network algorithms or principal component analyses are applied and have been used in separating training groups with known disease. Testing for unknown samples has not been very successful yet [24,25]. Much more convincing results were obtained by Scott-Thomas et al. [26], where 2-aminoacetophenone (2-AA) was measured by gas chromatography mass spectrometry (GC-MS) in cystic fibrosis patients (CF) as volatile biomarker produced by *P. aeruginosa*, which was also confirmed by other researchers [27-30]. Significantly higher levels of 2-aminoacetophenone were found in exhaled breath of cystic fibrosis patients colonized with *P. aeruginosa* while the concentration of this metabolite was below the detection limit in both control groups (healthy subjects and CF patients colonized with other bacteria species). Encouraging results were obtained in other studies, where specific volatile biomarkers of *Aspergillus spp.* were detected in exhaled breath of tuberculosis patients colonized with *Mycobacterium tuberculosis* cells [21,31-34].

The aim of this study is to characterize the release or consumption of VOCs by *S. aureus* and *P. aeruginosa.* Headspace samples from cultures of both pathogens were collected and preconcentrated on multibed sorption tubes and analyzed by GC-MS. Sampling was done under strictly controlled ventilation conditions at several time points to follow the dynamic changes in temporal VOC concentration profiles.

## Results

### GC-MS analysis

The initial amount of cells in the fermenters amounted to 4.04 × 10^5^ ± 2.75 × 10^5^ colony forming units (CFUs*ml^-1^) for *S. aureus* (n = 5) and 2.2 × 10^6^ ±5.1 × 10^5^ CFUs*ml^-1^ for *P. aeruginosa* (n = 7). The average cell densities and ODs at 600 nm are presented in Table 1. For both tested species all headspace samples were collected within the logarithmic phase of microbial proliferation. Importantly, bronchoalveolar lavage (BAL) studies have typically used a diagnostic threshold of 10^4^ or 10^5^ CFU/ml to define both the presence of pneumonia and the etiologic pathogen [35]. Naturally, the real number of bacteria in lungs is higher since a 10-fold dilution of BAL samples and the large surface area of the alveolar zone have to be considered.

**Table 1:** Average CFUs*ml^**-1**^**and OD**_**600**_**of*S. aureus*cultures at different incubation times**

	cfu*ml^-1^				**optical density 600 nm**			
	***S. aureus***		***P. aeruginosa***		***S. aureus***		***P. aeruginosa***	
**Time point**	**average**	**stdev**	**average**	**stdev**	**average**	**stdev**	**average**	**stdev**
0 h	4.04E + 5	2.75E + 5	2.17E + 06	5.13E + 05	0.0291	0.0134	0.047	0.008
1 h 30 m	2.38E + 6	1.63E + 6	9.76E + 06	3.33E + 06	0.0349	0.0111	0.051	0.005
2 h 15 m	-	-	1,83E + 07	6.13E + 06	-	-	0.058	0.005
3 h 00 m	7.38E + 6	3.73E + 6	6.17E + 07	2.33E + 07	0.0652	0.0076	0.066	0.005
3 h 45 m	-	-	1.18E + 08	6.32E + 07	-	-	0.077	0.012
4 h 30 m	4.95E + 7	2.91E + 7	1.61E + 08	7.35E + 07	0.1814	0.0190	0.088	0.012
5 h 15 m	-	-	1.83E + 08	8.12E + 07	-	-	0.097	0.012
6 h 00 m	1.30E + 8	4.52E + 7	2.91E + 08	1.19E + 08	0.2531	0.0085	0.101	0.015
24 h 00 m	-	-	2.31E + 09	1.02E + 09	-	-	0.511	0.138
26 h 00 m	-	-	4.64E + 09	1.35E + 09	-	-	0.813	0.133
28 h 00 m	-	-	5.91E + 09	2.46E + 09	-	-	0.892	0.109

**Figure 1:**
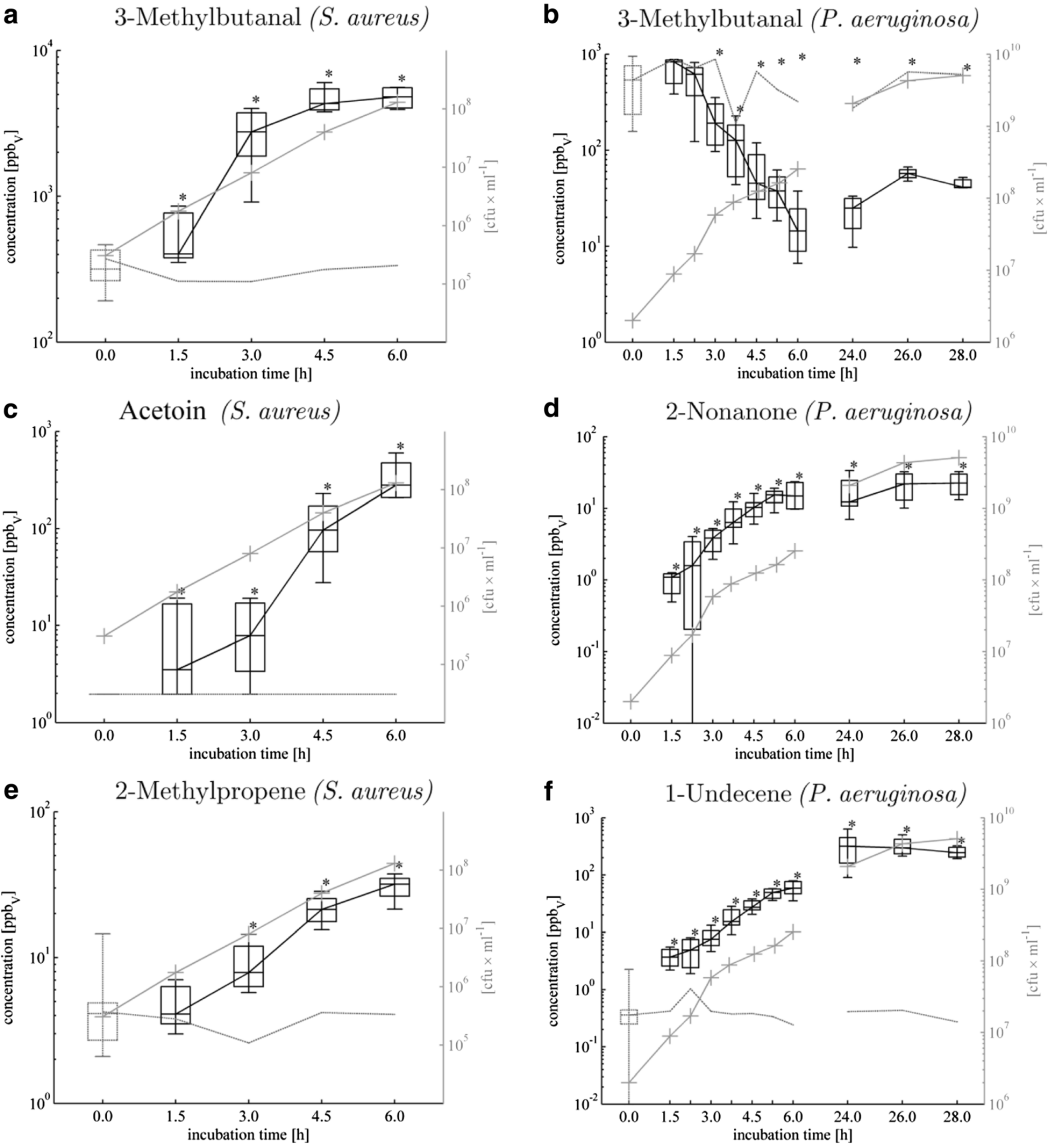
**Time-dependent a-f: Release (respectively consumption) of exemplary VOCs from*S. aureus*and*P. aeruginosa.*** Determined median concentrations [ppb_v_] with 25^th^ and 75^th^ percentiles [ppb_v_] are given as black boxes with whiskers indicating 5^th^ and 95^th^ percentiles and analogous gray box with gray line without markers indicates medium control. Gray lines with crosses denotes proliferation rate [CFUs*ml^-1^]. P-values <0,05 calculated by means of Kruskal-Wallis test indicate significant differences of controls compared to bacteria cultures.

A high number of different VOCs were found to be released by both bacterial species in a concentration range varying from part per trillion (ppt_v_) to part per million (ppm_v_). Also several volatile compounds were consumed by the bacteria, particularly by *P. aeruginosa* (Tables 2 and 3B, Figure 1b). *S. aureus* released 32 VOCs of diverse chemical classes amongst which 28 were analyzed in Selected Ion Monitoring mode (SIM) and 4 in Total Ion Chromatogram mode (TIC), comprising 9 aldehydes, 4 alcohols, 3 ketones, 2 acids, 2 sulphur containing compounds, 6 esters and 6 hydrocarbons. Only benzaldehyde was found in decreased concentrations in *S. aureus* cultures compared to medium controls.

**Table 2:** **Median concentrations of VOCs released or consumed by*Staphylococcus aureus***

			**median concentrations [ppbv]**				
**Compound**	**CAS**	**m/z for SIM**	**medium**	**1.5 h**	**3.0 h**	**4.5 h**	**6.0 h**
propanal	123-38-6	57	3.955	***10.62***	***14.22***	***8.932***	***7.04***
3-methyl-2-butenal	107-86-8	55, 84	1.526	1.832	***3.415***	***5.708***	***5.348***
2-ethylacrolein	922-63-4	84	1.656	2.01	***6.453***	***5.537***	***5.775***
(Z)-2-methyl-2-butenal	1115-11-3	84	73.48	81.91	***177.4***	***268.5***	***247.9***
(E)-2-methyl-2-butenal	497-03-0	84	< LOD	< LOD	***0.259***	***0.394***	***0.381***
benzaldehyde ^§^	100-52-7	107	20.64	19.08	17.65	**12.66**	**3.815**
methacrolein	78-85-3	70	5.922	5.644	***9.328***	7.617	6.36
acetaldehyde	75-07-0	43	528.5	606.4	***374.2***	***1022.7***	***1417.4***
3-methylbutanal **	590-86-3	**-**	317.1	***403.3***	***2764.3***	***4779.3***	***4818.5***
2-methylpropanal **	78-84-2	_**−**_	598.6	658.5	***2044.5***	***1698.6***	***1299.5***
1-butanol	71-36-3	56	< LOD	< LOD	< LOD	***21.24***	***59.4***
2-methyl-1-propanol	78-83-1	56, 74	0	0	0	***21.32***	***52.62***
3-methyl-1-butanol	123-51-3	55, 70	0	0	0	***27.65***	***210.0***
ethanol **	64-17-5	**-**	0	***89.57***	***237.0***	***6173.0***	***11695.1***
acetoin (hydroxybutanone)	513-86-0	88	< LOD	_***3.59***_	_***8.004***_	***140.6***	***279.3***
acetol (hydroxyacetone)	116-09-6	74	< LOD	< LOD	< LOD	***113.5***	***331.0***
2,3-butanedione	431-03-8	86	22.65	23.92	27.45	***49.84***	***67.99***
acetic acid	64-19-7	45, 60	0	0	0	***880.5***	***2566.6***
isovaleric acid	503-74-2	60	0	0	0	***31.13***	***97.35***
ethyl acetate	141-78-6	61	0	0	0	***1.973***	***5.624***
*n*-butyl acetate	123-86-4	56, 73	0	0	0	***0***	***0.239***
ethyl isovalerate	108-64-5	70	0	0	0	< LOD	***0.852***
isopentyl acetate	123-92-2	55, 70	0	0	0	< LOD	***1.938***
ethyl formate	109-94-4	31	0	0	0	< LOD	***3.188***
methyl methacrylate **	80-62-6	**-**	15.99	14.79	20.27	***28.65***	***31.93***
methanethiol	74-93-1	47	134.2	***210.4***	***360.6***	***559.4***	***701.5***
dimethyldisulfide (DMDS)	624-92-0	94	1.558	2.221	***3.657***	***8.134***	***10.24***
1,3-butadiene	106-99-0	54	< LOD	< LOD	***4.941***	***4.342***	***4.313***
2-methylpropene	115-11-7	56	< LOD	< LOD	***4.546***	***14.31***	***21.89***
n-butane	106-97-8	58	0.664	0.703	***1.274***	***2.504***	***4.329***
(Z)-2-butene	590-18-1	56	0	0	< LOD	***3.687***	***4.789***
(E)-2-butene	624-64-6	56	1.344	< LOD	***4.793***	***11.32***	***13.73***
propane	74-98-6	43, 41	0.91	0.815	1.951	***3.441***	***4.902***

Bold numbers indicate significant difference (Kruskal-Wallis test) in VOC concentrations between bacteria cultures and medium headspace (p < 0.05). Ethanol, 2-methylpropanal, 3- methylbutanal and methyl methacrylate were analyzed in TIC mode as indicated by **, while the remaining compounds were analyzed in SIM mode. Number of independent experiments n = 5 for each time point of bacteria growth, n = 14 for all medium controls. Concentrations are given in ppb_v_, ^§^ uptake (decreased concentration).

**Table 3:** **A and B: Median concentrations of VOCs released (A) or taken up (B) by*Pseudomonas aeruginosa***

**Compound**	**CAS**	**m/z for SIM**	**M [ppbv]**	**1.5 (n = 3)**	**2.25 (n = 4)**	**3 (n = 4)**	**3.75 (n = 5)**	**4.5 (n = 5)**	**5.20 (n = 4)**	**6 (n = 6)**	**24 (n = 5)**	**26 (n = 4)**	**28 (n = 3)**
**A)**													
3-methyl-1-butanol	123-51-3	55, 70	<LOD	<LOD	<LOD	<LOD	<LOD	<LOD	<LOD	<LOD	***62.56***	***148.4***	***142.2***
ethanol*	64-17-5	**-**	102.1	***623.5***	***322.2***	***396.4***	***441.4***	***548.9***	***800.0***	***761.6***	203.1	***333.3***	***350.4***
2-butanol^#^	78-92-2	45	0	0	0	0	0	0	0	0	0	***1.5E + 04***	***8.5E + 03***
2-nonanone	821-55-6	43, 56, 71	<LOD	***1.091***	***1.586***	***3.855***	***6.372***	***10.29***	***15.33***	***14.83***	***12.24***	***21.82***	***22.42***
2-pentanone	107-87-9	43, 86	<LOD	<LOD	<LOD	<LOD	<LOD	***0.526***	***0.910***	***0.901***	***12.91***	***19.30***	***17.94***
2-heptanone	110-43-0	43, 71	n.d.	<LOD	<LOD	<LOD	<LOD	<LOD	***0.286***	***0.259***	***2.700***	***4.789***	***3.622***
4-heptanone	123-19-3	43, 71	n.d.	n.d.	n.d.	n.d.	n.d.	<LOD	***0.422***	***0.496***	***1.000***	***2.079***	***1.088***
3-octanone*	106-68-3	**-**	n.d.	n.d.	n.d.	n.d.	n.d.	n.d.	n.d.	<LOD	***0.557***	***0.817***	<LOD
2-butanone*	78-93-3	**-**	10.08	***25.49***	***23.57***	15.89	17.90	17.11	***19.39***	14.65	30.39	***40.55***	***40.03***
methyl isobutyl ketone^#^	108-10-1	85, 100	3.8E + 04	8.7E + 04	8.0E + 04	5.5E + 04	***7.9E + 04***	6.5E + 04	***7.6E + 04***	6.4E + 04	***2.3E + 05***	***3.8E + 05***	2.7E + 05
ethyl acetate	141-78-6	61	<LOD	***1.936***	1.123	***0.777***	***1.556***	***1.167***	***1.088***	***1.231***	***1.972***	***2.686***	***1.895***
methyl 2-methylbutyrate	868-57-5	56, 85	n.d.	n.d.	n.d.	n.d.	n.d.	n.d.	n.d.	n.d.	n.d.	***0.637***	***1.669***
methyl methacrylate*	80-62-6	**-**	24.81	***38.14***	***44.49***	32.28	***44.03***	***36.81***	***46.67***	***38.67***	47.72	54.17	48.13
ethyl 2-methylbutyrate^#^	7452-79-1	57, 74, 85	0	0	0	0	0	0	0	0	***7.5E + 04***	***1.4E + 05***	***1.8E + 05***
2-methylbutyl isobutyrate^#^	2445-69-4	55, 70	0	0	0	0	0	0	0	0	***5.2E + 05***	***1.2E + 06***	***1.3E + 06***
isoamyl butyrate^#^	106-27-4	43, 71	0	0	0	0	0	0	0	0	***2.5E + 05***	***1.4E + 06***	***7.6E + 05***
2-methylbutyl 2-methylbutyrate^#^	2445-78-5	57, 70, 85	0	0	0	0	0	0	0	0	***2.7E + 06***	***7.6E + 06***	***9.7E + 06***
amyl isovalerate^#^	25415-62-7	57, 70, 85	0	0	0	0	0	0	0	0	***1.9E + 06***	***3.9E + 06***	***6.1E + 06***
dimethyl sulfide (DMS)	75-18-3	47, 62	<LOD	<LOD	<LOD	n.d.	<LOD	<LOD	<LOD	***1.295***	***99.54***	***143.0***	***173.6***
Dimethyl disulfide (DMDS)	624-92-0	94	0.580	**1.817**	1.042	0.663	0.605	0.538	0.600	0.597	***5.909***	***14.11***	***11.09***
dimethyl trisulfide (DMTS)	3658-80-8	126	<LOD	<LOD	<LOD	<LOD	<LOD	<LOD	<LOD	<LOD	***0.324***	***0.764***	***1.106***
methanethiol	74-93-1	47	33.03	45.55	47.77	21.86	21.31	18.22	25.25	24.64	***261.2***	***418.0***	***318.1***
mercaptoacetone^#^	24653-75-6	90	0	0	0	0	0	0	0	0	***1.7E + 05***	***2.6E + 05***	***2.1E + 05***
2-methoxy-5-methylthiophene^#^	31053-55-1	113	0	0	0	0	0	0	0	0	***1.1E + 06***	***2.0E + 06***	***1.6E + 06***
3-(ethylthio)-propanal^#^	5454-45-5	62	0	0	0	0	0	0	0	0	***5.1E + 04***	***3.2E + 05***	***7.9E + 05***
1-undecene	821-95-4	41, 55, 69	0.337	***3.687***	***4.891***	***7.566***	***15.30***	***27.24***	***49.10***	***58.73***	***317.5***	***296.1***	***245.0***
2-methyl-2-butene	513-35-9	55, 70	<LOD	<LOD	<LOD	***0.138***	***0.221***	***0.324***	***0.492***	***0.651***	***0.524***	***0.512***	***0.406***
1,10-undecadiene	13688-67-0	41, 55, 69	<LOD	<LOD	<LOD	<LOD	<LOD	***0.516***	***0.838***	***0.993***	***6.813***	***6.349***	***4.515***
1-nonene	124-11-8	55, 70, 126	0.269	0.419	0.336	0.299	***0.370***	***0.419***	***0.541***	***0.588***	***2.613***	***3.401***	***2.623***
1-decene	872-05-9	55, 70	<LOD	<LOD	0.283	0.207	0.203	0.221	***0.289***	***0.325***	***1.178***	***1.213***	***0.910***
1-dodecene	112-41-4	57, 70, 85	1.861	4.596	3.341	2.211	3.221	2.017	3.148	***2.646***	***9.494***	***9.129***	***8.242***
butane	106-97-8	58	0.331	0.471	0.283	0.160	0.143	0.154	0.275	***0.184***	***0.673***	***1.482***	***1.400***
isoprene*	78-79-5	**-**	<LOD	***2.110***	***3.156***	***7.121***	***10.28***	***12.25***	***14.77***	***16.80***	***20.40***	***20.09***	***12.47***
10-methyl-1-undecene#	22370-55-4	57, 70, 85	0	0	0	0	0	0	0	0	***3.3E + 05***	***3.2E + 05***	***2.9E + 05***
pyrrole	109-97-7	41, 67	1.105	***29.62***	***48.16***	***49.66***	***39.84***	***20.50***	***22.59***	***13.12***	***15.55***	***21.01***	***17.50***
3-methylpyrrole*	616-43-3	**-**	<LOD	<LOD	<LOD	<LOD	<LOD	<LOD	***5.272***	***8.278***	***24.74***	***24.57***	***18.92***
1-vinyl aziridine^#^	5628-99-9	41, 67	0	***2.3E + 07***	***2.8E + 07***	***2.1E + 07***	***1.1E + 07***	***4.8E + 06***	***3.5E + 06***	***1.1E + 06***	***5.0E + 04***	***4.6E + 05***	0
**B)**													
butanedione	431-03-8	86	77.22	122.9	112.9	57.27	50.76	***24.49***	***22.30***	***9.568***	***5.131***	***7.535***	***8.746***
benzaldehyde	100-52-7	107	183.9	145.2	102.2	***26.50***	***13.11***	***9.944***	***9.434***	***7.024***	***5.698***	***7.082***	***8.538***
acetaldehyde	75-07-0	43	515.5	340.6	316.1	***65.15***	***47.75***	***53.22***	***87.89***	***87.14***	***30.84***	***42.56***	***22.97***
methacroleian	78-85-3	70	3.291	4.175	3.237	***0.922***	***0.502***	***0.209***	***0.187***	***<LOD***	***<LOD***	***<LOD***	***<LOD***
3-methylbutanal*	590-86-3	**-**	419.6	832.1	620.1	***191.3***	***126.8***	***45.23***	***37.63***	***14.52***	***24.89***	***57.25***	***41.17***
nonanal	124-19-6	43, 58, 71	13.44	9.317	8.969	***6.332***	***7.285***	***7.379***	7.397	***6.608***	***4.122***	6.176	***6.222***
propanal	123-38-6	57	2.944	3.382	2.222	***0.958***	***1.132***	***0.967***	1.112	***0.863***	***<LOD***	***<LOD***	***<LOD***
3-methyl-2-butenal	107-86-8	55, 84	1.266	1.578	1.617	0.953	0.856	0.641	***0.515***	***<LOD***	***<LOD***	***<LOD***	***n.d.***
acrolein	107-02-8	56	9.951	7.257	11.23	9.622	6.918	7.082	9.965	7.432	***4.036***	***3.915***	***3.628***
butanal*	123-72-8	**-**	24.35	22.71	11.00	***1.305***	***<LOD***	***1.129***	***1.837***	***2.259***	***<LOD***	***<LOD***	***<LOD***
2-methylpropanal*	78-84-2	**-**	181.9	***273.3***	199.8	80.03	***28.30***	***11.41***	***7.520***	***4.378***	***4.057***	***6.026***	***4.851***
octanal*	124-13-0	**-**	5.424	4.226	4.282	3.410	***2.448***	***2.507***	3.011	***1.791***	***1.266***	1.950	***2.580***

Bold numbers indicate significant difference (Kruskal-Wallis test) between VOC concentrations in bacteria cultures and medium (m) headspace (p < 0.05). * TIC mode analysis with determined concentrations (ppb_v_) and ^#^ SIM mode analysis semi-quantified by peak area, n=32 for medium control.

*P. aeruginosa* released 37 VOCs (32 VOCs analyzed in SIM mode and 5 VOCs analyzed in TIC mode) but mostly in lower amounts than *S. aureus*. Altogether 12 compounds were consumed by *P. aeruginosa* (9 VOCs analyzed in SIM mode and 3 VOCs analyzed in TIC mode), compared to only benzaldehyde consumed by *S. aureus*. The higher proliferation rates of *P. aeruginosa* cultures were found, and the respective CFU counts were still strongly increasing at the second day of incubation; hence the headspace sampling was performed also on day two after 24, 26 and 28 h of microbial growth. Six classes of compounds were found, comprising 9 hydrocarbons (8 with determined concentrations), 3 nitrogen containing compounds (2 with determined concentrations), 8 esters (3 with determined concentrations), 7 ketones (6 with determined concentrations), 7 sulphur containing compounds(4 with determined concentrations) and 3 alcohols (2 with determined concentrations). Decreased concentrations were measured for 12 compounds, including 11 aldehydes and 1 ketone (2,3-butanedione).

### Aldehydes

One of the most striking observations was the completely opposite behaviour with regard to this chemical class when comparing the two species: *S. aureus* released various aldehydes (Figure 1a), partly in high concentrations, while no release of aldehydes was observed for *P. aeruginosa*. (Table 3B, Figure 1b). Particularly 3-methylbutanal (Figure 1a), 2- methylpropanal, acetaldehyde and (Z)-2-methyl-2-butenal were found in strongly elevated concentrations in the headspace of *S. aureus* cultures. These four aldehydes increased to significant concentrations at early time points (1.5-3 h of incubation), hence at relatively low cell densities of the bacteria culture.

### Alcohols

Alcohols were produced by both bacteria species (Table 2 and 3A) and they were one of the most prominently released compounds in *S. aureus*. Especially ethanol was present in high concentrations at an early stage in the headspace of both bacteria. Besides, also 1-butanol, 2- methyl-1-propanol and 3-methyl-1-butanol were detected at significantly higher amounts at the earliest after 4.5 h of *S. aureus* growth. However, among the three alcohols released by *P. aeruginosa* only ethanol was present at significant levels on day one (<24 h since inoculation), while 3-methyl-1-butanol and 2-butanol reached significantly higher concentrations on the second day of the experiment.

### Ketones

Amongst three ketones released by *S. aureus*, acetoin (hydroxybutanone) (Figure 1c) and acetol (hydroxacetone) were found at higher concentration levels than 2,3-butanedione, which was observed at elevated concentration already in control samples. Acetoin was significantly released already after 1.5 h reaching high levels at 4.5 h and 6 h after inoculation, whereas the release of butanedione was weaker especially if the substantial background originating from the medium is considered.

Importantly, entirely different ketones were released by *P. aeruginosa*, comprising 2- butanone, 2-pentanone, methyl isobutyl ketone, 2-heptanone, 4-heptanone, 3-octanone and 2-nonanone (Figure 1d). Although they were found at relatively low concentrations, most of them were absent in medium controls (apart from 2-butanone and methyl isobutyl ketone).

With respect to breath gas analysis 2-nonanone is presumably the most interesting ketone released by *P. aeruginosa* due to its absence in medium controls and early significant appearance in bacteria cultures. Moreover, concentrations of 2-nonanone determined, correlated very well with the proliferation rate of *P. aeruginosa*.

### Acids and esters

Two acids were produced by *S. aureus*, isovaleric acid and acetic acid. Particularly prominent was the release of acetic acid, which reached over 2500 ppb_v_ (i.e. 2.5 ppm_v_) within only 6 h of bacterial growth (Table 2). It should be noted that none of these acids was found in the headspace of the medium controls. In contrast, no acids at all were released by *P. aeruginosa*.

All esters released by bacteria tested were detected in low concentrations and at relatively late time points with the exception of methyl methacrylate. Nevertheless, background concentrations of esters are comparatively high and not stable. Therefore, esters seem to have no value in breath analysis in infections caused by these pathogens.

### Volatile sulphur-containing compounds (VSCs)

Two volatile sulphur-containing compounds (VSCs) were found to be released from *S. aureus*, dimethyldisulfide (DMDS) and methanethiol (MeSH). The later one was detected at significantly higher concentrations already 1.5 h after inoculation and reached over 700ppb_v_ after 6 h of bacteria growth. Both VSCs were also released by *P. aeruginosa* but at substantially lower concentrations reaching ~0.6ppb_v_ of DMDS and ~25ppb_v_ of MeSH 6 h after inoculation (increased to ~11ppb_v_ and ~320ppb_v_, respectively, 28 h after inoculation).

Additionally, dimethylsulfide (DMS), dimethyltrisulfide (DMTS), mercaptoacetone, 3-(ethylthio)-propanal and 2-methoxy-5-methylthiophene were released by *P. aeruginosa* but at the earliest after 24 h of bacteria growth.

### Hydrocarbons

To our knowledge, low-molecular (C3 - C4) hydrocarbons as volatile metabolites released by pathogenic bacteria were not investigated so far. This is mostly due to technical reasons, it is impossible to analyse these compounds by direct spectrometric techniques like PTR-MS, while most of GC-MS analyses were done so far with solid phase microextraction (SPME) or sorption tubes filled with Tenax only. Tenax is not suitable to adsorb as low molecular hydrocarbons as C3 and gives very poor adsorption efficiency for C4 [36]. Therefore multibed sorption tubes were applied in the present work within which carbon molecular sieves (Carboxen 569 and Carboxen 1000) very efficiently trap the most volatile analytes (propane, butane). Consequently, the analyses of these compounds were performed at the trace level, giving the limit of detection (LOD) for propane at 33ppt_v_ and for butane 24ppt_v_ (data not shown).

**Figure 2:**
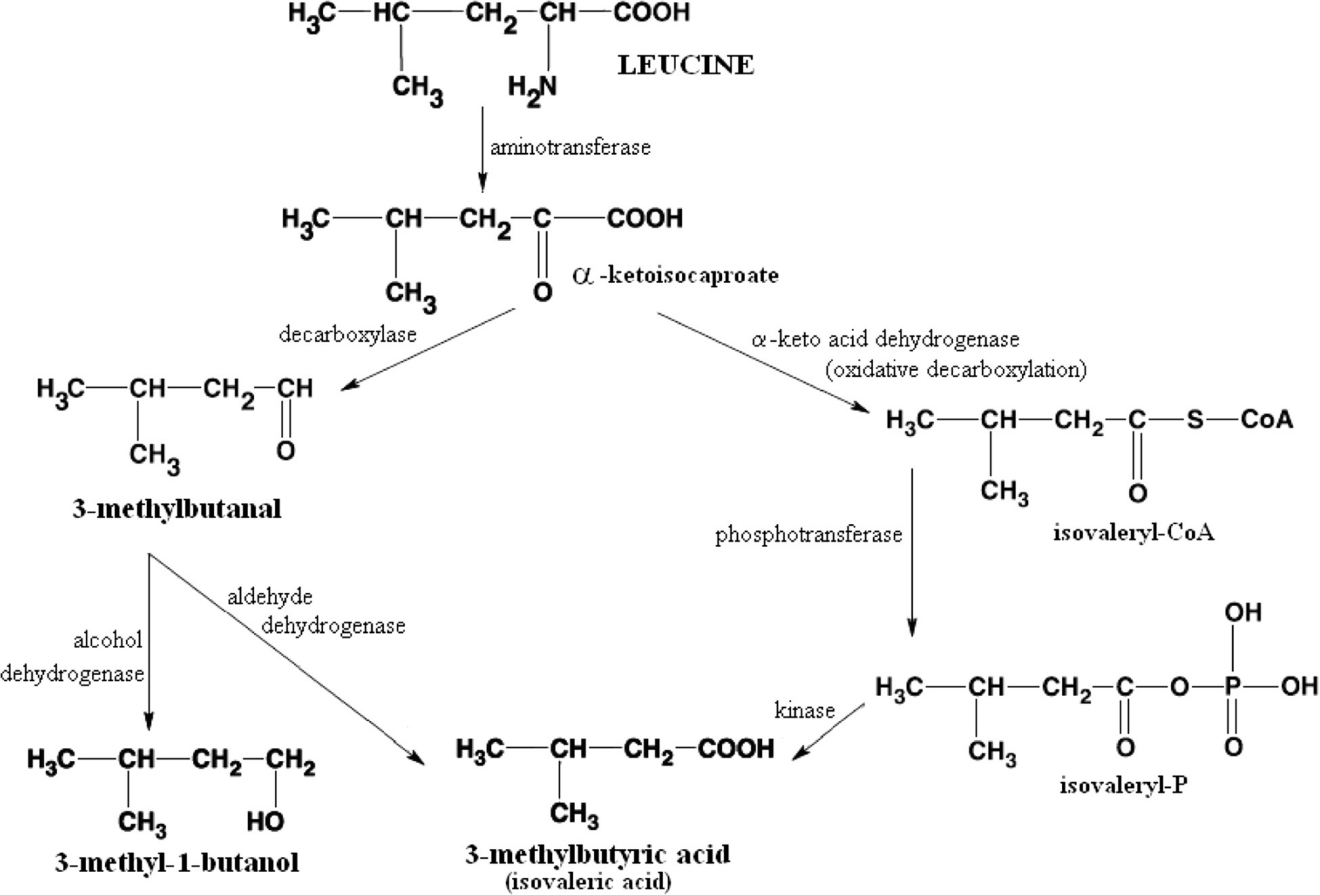
**Catabolism of leucine leads to the formation of 3-methylbutanal, 3-methyl-1-butanol and 3-methylbutyric acid (isovaleric acid), which were found to be significantly released by*S. aureus*.** Modified after Marilley et al. [19]

Diverse hydrocarbons were detected mostly in low amount in the headspace of *S. aureus* and *P. aeruginosa* cultures comprising 6 and 9 different compounds, respectively. Concerning *S. aureus* solely 2-methylpropene (Figure 1e) and (E)-2-butene reached moderately high concentration levels. Intriguingly, all hydrocarbons released by *S. aureus* consist of 4 carbon atoms (except propane) while *P. aeruginosa* released larger alkenes mostly in the range of C9 - C12. Amongst all volatile metabolites released from *P. aeruginosa* hydrocarbons were one of the most important chemical classes. In particular, 1-undecene and isoprene were significantly released already at the first sampling time-point, reaching as high concentration as ~300ppb_v_ after 24 h of bacteria growth. Importantly concentrations of 1-undecene in headspace samples were very well correlated with the proliferation rate of *P. aeruginosa* (Figure 1f). Isoprene, the second most abundant hydrocarbon secreted by *P. aeruginosa* whose biosynthesis via methylerythritol phosphate (MEP) pathway was found in a wide range of plants and microorganisms [37,38] reached the maximum concentration of 24 ppb_v_ after 24 h of bacteria growth. All remaining hydrocarbons were detected at low (e.g. 1-dodecene) or even extremely low concentration (e.g. 2-methyl-2-butene, 1-decene in Table 3A).

### Volatile nitrogen-containing compounds (VNCs)

A smaller, but very interesting class of compounds exclusively released by *P. aeruginosa* comprised volatile nitrogen containing compounds (VNCs). The preeminent example is pyrrole, which was detected already after 1.5 h and reached the maximum concentration of ~50ppb_v_ after 3 h of bacteria growth. Interestingly, apart from 3-methylpyrrole, the VNCs had an unconventional pattern of release, reaching the maximum concentration at early time-points and continuously decreasing in the course of experiment, while they were absent in the medium control.

## Discussion

The aim of this work was to investigate whether the detection and perhaps identification of bacteria can be achieved by the determination of characteristic volatile metabolites released. This work should provide the basis for the application of breath-gas analysis in the early and non-invasive diagnosis of bacterial lung infections by monitoring the presence of the specific pathogen-derived markers in exhaled breath. Exhaled breath analysis is a relatively new field of research and still requires extensive basic research to evaluate the candidate compounds, which may serve as biomarkers, and to delineate their possible biochemical and cellular sources. Volatile compounds in exhaled breath may be of endogenous (i.e. derived from host cells), exogenous or microbial origin.

Hence it is crucial to investigate the contribution of microorganisms of the normal flora (originating from body compartments like the gut, upper airways, sinuses, nose or mouth) and of microorganisms expanded during infections to the VOCs found in human breath.

Numerous species which are found in normal flora of humans may also become pathogenic, e.g. when the immune system is weakened [2]. In this work two different bacterial species [2,39] were investigated with respect of the release of VOCs. In the past, such or similar investigations were performed applying GC-MS, however, mostly with only qualitative and not quantitative analysis of detected VOCs [6,7,9,10,26,40] or for instance with indirect quantification without calibration of VOCs of interest [30].

In our *in vitro* work we found that the patterns of VOC release from *S. aureus* and *P. aeruginosa* are only in part identical, and considerable differences were found concerning the dynamics of VOC production and especially the uptake of volatile metabolites. Thus, *P. aeruginosa* takes up or catabolizes (but never releases) aldehydes, in contrast to *S. aureus*, which releases high concentrations of aldehydes. Similarly, no acids were significantly released by *P. aeruginosa* in our study. Despite higher proliferation rate of *P. aeruginosa* the concentrations of released metabolites were lower from those secreted by *S. aureus.* A greater variety of volatile compounds was found in the headspace of *P. aeruginosa* as compared to *S. aureus* comprising diverse ketones, esters, sulfur containing compounds, hydrocarbons and additionally nitrogen containing compounds, which were not detectable in the headspace of *S. aureus*.

Zechman and co-workers have identified several identical compounds as reported here in the headspace of *S. aureus* and *P. aeruginosa* (e.g. acetoin and methylbutanal for *S. aureus*, 1-undecene and ketones for *P. aeruginosa* and DMDS and *iso*-pentanol for both species) using aerobic conditions similar to us with application of liquid culture and tryptic soy broth as culture medium [6]. However, they did only qualitative analyses at one incubation time point of 24 h. Besides similarities in our study to other works, also divergent results were obtained [6,7,11,26,30,40]. In this respect, Scott-Thomas [26] and Labows [30] identified 2-aminoacetophenone as an important volatile metabolite of *P. aeruginosa,* which allows discrimination of cystic fibrosis patients colonized with *P. aeruginosa* from control groups (healthy subjects and CF patients colonized with other bacteria species) [26]. This compound could not be detected in the headspace of *P. aeruginosa* in our study since it is derived from tryptophan, whose concentration might be too low in the used soy based medium to form aminoacetophenone in detectable concentrations. Similarly, Allardyce et al. reported strong release of acetic acid and acetaldehyde from *P. aeruginosa*[11], whereas acetaldehyde was clearly decreasing in the *Pseudomonas* cultures in our study. Presumably, culture conditions (especially nutrient availability) and analytical methodologies may have a strong influence on the release of VOCs from bacteria cells, stressing the importance to standardize these factors.

Although it might be insufficient to reveal the full spectrum of potential volatile metabolites, a single growth medium (tryptic soy broth) was used for bacteria cultivation in our experiments. This medium is standard for bacteria culture ensuring fast proliferation of microorganisms. Standardization of culture conditions (e.g. proposed here application of the same medium for both species) will be a challenge for the future as bacteria differ in their requirements for nutrients and the composition of the medium in use may affect the nature of the compounds released.

The sampling of headspace gas was performed at several different time points to gain insight into the dynamics of microbial VOC production. This approach demonstrated varying VOC concentration profiles. Accurate diagnosis will require knowledge at what time after inoculation volatile metabolites show either maximum release or become steady in concentration. Although this study was limited to two species we observed specific VOC patterns for each bacterium, demonstrating the procedure developed is suitable to discriminate between pathogenic bacteria. An important issue which should be addressed in future studies is to gain insight into the VOC profiles of further clinically relevant microorganisms and to address the effect of the presence of additional pathogenic organisms in the samples as well as of the presence of host cells.

The metabolic origin of VOCs released is not completely elucidated but it is known that production of branched-chain aldehydes results from the catabolism of amino acid (Figure 2) [19,41-43]. Aldehydes then can be reduced to alcohols by alcohol dehydrogenases (e.g. 3-methylbutanal to 3-methyl-1-butanol) or oxidized to carboxylic acids by an aldehyde dehydrogenase (e.g. 3-methylbutanal to isovaleric acid) as observed for *S. aureus*. Since all aforementioned compounds were found to be released by *S. aureus* in our *in vitro* study we presume that amino acid degradation rather than synthesis of fatty acids from alkanes is the underlying pattern of VOCs released by *S. aureus*, especially since the culture medium used in our experiments consisted mainly of amino acids, peptides and glucose. This hypothesis is also supported by other published work, where a link between availability of branched amino acids (e.g. valine, isolecine) and production of branched alcohols and aldehydes was reported [6].

**Figure 3:**
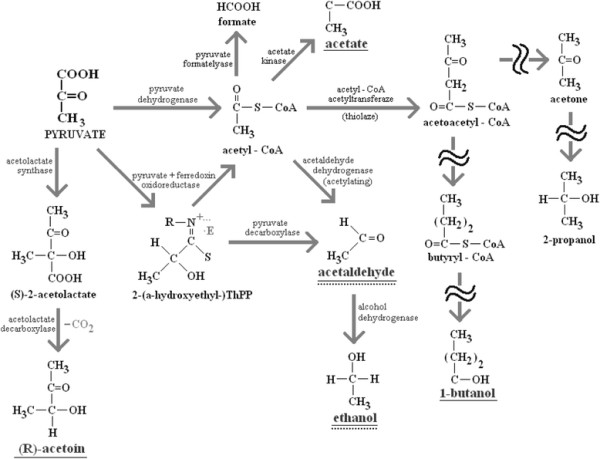
**Simplified scheme of pyruvate metabolism via glycolytic fermentations and lactate converting fermentations, modified after Michal et al.**[44]**.** Exclusively, pathways which lead to the production of VOCs significantly released by *S. aureus* in this study (underlined with solid line) are presented, including acetoin (3-hydroxy-2-butanone), acetaldehyde, ethanol, 1-butanol, acetone, 2-propanol. In case of *P. aeruginosa* the metabolism of amino acids rather than glycolysis of carbohydrates yields pyruvate as starting material (significantly released or taken up products are underlined with dotted line).

Additionally, pyruvate or citrate are starting materials for the formation of short-chain flavor compounds such as acetoin, 2,3-butanedione, 1-butanol, 2-propanol, acetic acid, acetaldehyde and ethanol through glycolytic, lactate converting and non-glycolytic carbohydrates fermentations or fermentations of nitrogenous compounds [44]. The catabolism of pyruvate (presented on Figure 3) seems to play an important role in case of *S. aureus* since the products of this metabolic pathway were found in the headspace of this bacterium in our study and also by other researchers, inter alia ethanol, acetaldehyde, acetic acid [11] and acetoin [6,40].

Detailed investigation of the subspecies of the genus *Staphylococcus* shows that acetoin is produced by the subspecies *aureus* and not by the subspecies *anaerobius*. On the other hand, *Pseudomonads* are described as organisms with strictly respiratory metabolism mostly with oxygen and in some species nitrate as terminal electron acceptor [45], hence the release of alcohols and acids from these microorganisms is not expected. Indeed, carboxylic acids were not observed to be released by *P. aeruginosa* in our *in vitro* study, but a very week production of 2-butanol and substantially stronger of ethanol and 3-methyl-1-butanol were found. These may be related to altered activity of aldehyde- and alcoholdehydrogenase as reported by Nosova et al. [46] while the metabolism of amino acids [44] rather than glycolysis of carbohydrates via Entner-Doudoroff pathway [1] yields pyruvate as starting material under conditions applied in our study. Nevertheless, it seems that the most dominant metabolic process in *P. aeruginosa* cultures is the catabolism of organic compounds such as aldehydes as carbon and energy sources. The versatile nutritional requirements of *Pseudomonas* are commonly known and some of its subspecies utilize over 100 different compounds of diverse chemical classes what makes them particularly important organisms of bioremediation in environment (degradation of oil spills, pesticides and other xenobiotics) [1,47]. In respect to this feature, the substantial uptake of aldehydes from culture medium was observed in experiments with *P. aeruginosa*, while acetaldehyde, 3-methylbutanal, 2-methylpropanal, benzaldehyde and butanal were most strongest metabolized.

Our results confirm the production of sulfur-containing compounds, especially by *P. aeruginosa*, extending the earlier works of other researchers [6,7,30]. VSCs such as dimethylsulfide, dimethyldisulfide and dimethyltrisulfide originate from auto-oxidation of methanethiol [19,48,49] that can be produced though metabolism of the sulfur-containing amino acids, e.g. via demethiolation [50], transamination [51-53] or recombination pathway [54].

One of the most interesting observations in experiments with *P. aeruginosa* is the early and strong release of the nitrogen containing compounds pyrrole, 1-vinyl aziridine and 3-methylpyrrole with aberrant release patterns concerning the first two mentioned compounds compared to all other released metabolites. This finding is unique among tested bacteria species and particularly interesting from the point of view of early detection of *P. aeruginosa* infections.

Both investigated bacteria release in part the same compounds, mostly alcohols, esters and VSCs (Tables 2 and 3). As such, these compounds cannot be used for an unambiguous identification of the underlying pathogen. However, they can be used in exhaled breath analysis to monitor development of disease (e.g. emerging pneumonia), especially that some of them are released at as high concentration levels as several hundreds of ppb_v_ (e.g. methanethiol, 3-methyl-1-butanol). Nevertheless, both bacteria *S. aureus* and *P. aeruginosa* normally do not coexist as the pathogens of pneumonia. In addition, our *in vitro* study clearly shows that both bacteria produce pathogen-specific metabolites allowing their identification by means of gas phase analysis. VOCs exclusively released by *S. aureus* comprise mostly low molecular weight analytes, while the compounds within the range of C3 - C5 have the biggest contribution, being 76% of all *unique* metabolites for this bacterium. Similarly, there is a set of metabolites exclusively released by *P. aeruginosa*. Several compounds show significantly increased concentrations already in the first few hours of bacterial growth. Among them, nitrogen-containing VOCs were released early after incubation of *P. aeruginosa*, but also ketones (besides methyl isobutyl ketone) and most of unsaturated hydrocarbons.

Compounds like acetone, isoprene, acetaldehyde and butane are normally present in human breath [55-60] resulting in substantially high background level and therefore they are unsuitable as biomarkers. We propose a candidate compound should not be present in more than 5% of healthy non-smoking subjects, ideally. Volatile metabolites fulfilling our criteria are listed in Table 4. In this respect, particularly intriguing substances are nitrogen-containing metabolites such as 1-vinylaziridine and 3-methylpyrrole, which are increasing strongly during the first incubation phase of *P. aeruginosa* and have a decreasing tendency afterwards.

**Table 4:** **Maximum median concentrations [ppb**_**v**_**] with respective time of bacteria growth [h] as well as appearance in exhaled breath of healthy volunteers for selected metabolites which fulfill the criteria for biomarker of*Staphylococcus aureus*and*Pseudomonas aeruginosa*(based on*in vitro*experiments)**

**Compound**	**Staphylococcus aureus**			**Pseudomonas aeruginosa**			**occurrence [%] in healthy NON-smokers**	**occurrence [%] in healthy smokers**
	**max. conc. [ppb**_v_**]**	**growth time for max. conc.**	**growth time for 1st significant increase**	**max. conc. [ppb**_v_**]**	**growth time for max. conc.**	**growth time for 1st significant increase**		
**2-nonanone**	n. s.	-		22.4	28 h	1 h 30 min	**0**	**0**
**1-nonene**	n. s.	-		3.4	26 h	3 h 45 min	**0**	**0**
**1-decene**	n. s.	-		1.2	26 h	5 h 20 min	**0**	**0**
**1,10-undecadiene**	n. s.	-		6.8	24 h	4 h 30 min	**0**	**0**
**1-dodecene**	n. s.	-		9.5	24 h	6 h	**0**	5,6
**1-undecene**	n. s.	-		317.5	24 h	1 h 30 min	**0**	5,6
**1-vinylaziridine**	n. s.	-		2.8E + 07	2 h 15 min	1 h 30 min	**0**	**0**
**3-methylpyrrole**	n. s.	-		24.74	24 h	5 h 20 min	**3,6**	**0**
**acetol**	331.0	6 h	4 h 30 min	n. s.	-	-	**0**	**0**
**acetoin**	279.3	6 h	1 h 30 min	n. s.	-	-	**3,6**	**0**
**(E)-2-butene**	13.73	6 h	3 h	n. s.	-	-	**0**	11,1
**(Z)-2-butene**	4.789	6 h	4 h 30 min	n. s.	-	-	**0**	5,6
**1-butanol**	59.40	6 h	4 h 30 min	n. s.	-	-	**0**	**0**
**ethyl formate**	3.188	6 h	6 h	n. s.	-	-	**0**	**0**
**isopentyl acetate**	1.938	6 h	6 h	n. s.	-	-	**0**	**0**
**ethyl isovalerate**	0.852	6 h	6 h	n. s.	-	-	**0**	**0**
**2-ethylacrolein**	6.453	3 h	3 h	n. s.	-	-	**0**	**0**
**(Z)-2-methyl-2-butenal**	268.5	4 h 30 min	3 h	n. s.	-	-	**0**	**0**
**isovaleric acid**	97.35	6 h	4 h 30 min	n. s.	-	-	**0**	5,6

1-Vinylaziridine is exclusively given as peak area due to lack of commercially available standards. Populations of healthy subjects: n_smokers_ = 23, n_non-smokers_ = 32.

Very encouraging results were obtained also for α-unsaturated hydrocarbons, especially 1- undecene which was one of the most abundant VOCs produced by *P. aeruginosa*. 1-Undecene was significantly released from the first time-point of the experiment (1.5 h) and was never found in exhaled breath of healthy non-smokers. Interesting is also 2-nonanone, which was significantly released immediately after inoculation of *P. aeruginosa*, but never found in any exhaled breath sample. Similarly, acetoin and acetol meet all requirements for a perfect biomarker of *S. aureus*.

## Conclusions

In conclusion, the clear differences in the bacteria-specific profiles of VOC production were found, particularly with respect to aldehydes which were only taken up by *P. aeruginosa* and released by *S. aureus.* Considerable differences in VOCs profiles were observed also among ketones, hydrocarbons, alcohols, esters, VSCs and VNCs. The *in vitro* experiments were performed at bacterial densities which relate to the situation in the lungs of VAP patients, and the significant release of certain metabolites was found as early as 1.5 to 3 hours after inoculation of bacteria. Hence, our results give strong evidence that monitoring of pathogen- derived breath markers should enable non-invasive detection and perhaps even identification of virulent microorganisms causing VAP earlier than using conventional microbiological methods.

## Methods

### Bacteria cultivation

*Staphylococcus aureus* (ATCC 25923) and *Pseudomonas aeruginosa* (ATCC 27853) were investigated. Bacteria were inoculated in a 4 ml liquid preculture and grown over night at 37°C without agitation. Both species were cultivated in tryptic soy broth medium (Merck KGaA, Darmstadt, Germany) ensuring very fast proliferation rates for the purpose of bacteria’s headspace analysis by means of GC-MS. Plating for colony forming units (CFU) counts has been performed in duplicate on Mueller Hinton agar plates. 100 ml of medium in fermenters was inoculated by adding 100 μl of the preculture. As a control, tryptic soy broth medium was carried along and no other medium was tested for bacteria cultivation.

According to preliminary experiments headspace samples for GC-MS analysis were taken 1.5, 3, 4.5 and 6 h for *S. aureus*, respectively 1.5, 2.25, 3, 3.75, 4.5, 5.25, 6, 24, 26 and 28 h for *P. aeruginosa*. Aliquots for plating of the preculture were taken at t = 0 h and the remaining samples immediately prior to VOCs sampling time points. Samples were diluted 1:100 (10^-2^) or, if required, 1:10000 (10^-4^) in 0.9% NaCl and 50 μl of the dilutions were plated in duplicate on Mueller Hinten agar plates using an automated spiral plater (model WASP 2, Don Whithley, Shipley, UK), revealing a detection limit of 10^3^ CFU/ml. After overnight incubation at 37°C CFUs were determined. Additionally, photometric measurements of the optical density at 600 nm were performed at the indicated time points to monitor bacterial proliferation. For cultivation of bacteria a previously described device was used [61-64] allowing strictly controlled ventilation and VOC sampling from four independent cultures.

Dynamic headspace sampling with simultaneous preconcentration was performed by adsorption on multibed sorption tubes as described previously [61-64].

### GC-MS analysis

Composition of sorption tubes, conditions for bacteria headspace sampling, thermal desorption and calibrations as well as GC-MS settings are given elsewhere [61-64]. The temperature program of the chromatographic column was as follows: initial 55°C held for 6 min, then ramped 7°C/min up to 97°C (2 min), 2°C/min to 110°C (0 min), 5°C/min to 130°C (4 min), 5°C/min to 160°C (4 min), 4°C/min to 230°C (0 min) and 10°C/min to 280°C (4 min).

The constant helium flow rate of 1.8 ml/min was used as carrier gas. In addition to previous experiments, the mass spectrometer worked in a combined TIC*/*SIM mode. The TIC (total ion chromatogram), in the range of *m/z* 20 to *m/z* 200, was used for the identification of potential target compounds. Additionally, most of compounds were quantified using SIM (selective ion monitoring) mode with 100 ms dwell time for all ions used in SIM mode. Ions chosen for SIM monitoring of target analytes are given in Tables 2 and 3 along with the concentrations of VOCs released from bacteria cultures. The chromatographic data processing was performed by the Agilent Chemstation Software (GC-MS Data Analysis from Agilent, Waldbronn, Germany) while detected compounds were identified firstly by matching with the mass spectrum library NIST 2008 (Gaithersburg, MD, USA) and additionally confirmed with retention time of standardized reference materials. All compounds used for identification and quantification (calibration) were purchased from Sigma Aldrich (Sigma-Aldrich, Steinheim, Germany).

### Sampling procedure for human breath samples

A cohort of 55 individuals (32 non-smokers, 23 active-smokers) was recruited for this study. Amongst smokers, 12 males were in the age range from 22 to 64 years and 11 females were in the age range from 21 to 65 years. The cohort of non-smokers comprised 12 males and 20 females in the age range from 22 to 87 years. All individuals gave informed consent to their participation. The volunteers completed a questionnaire describing their current smoking status (active smokers, non-smokers, ex-smokers) and the time elapsed since last smoking (if applicable). No special dietary regimes were applied. All volunteers recruited to this study were healthy, especially in respect to lung diseases caused by bacterial infections but also asthma, chronic obstructive pulmonary disease (COPD) and lung cancer. The samples were collected at different times of the day at least 2 hours after last meal and were processed within 6 hours after sampling.

Volunteers were asked to rest for at least 5 minutes before sampling. The alveolar air samples were collected into Tedlar bags (SKC Inc, Eighty Four, PA) by means of an in-house produced breath sampler, allowing also the collection of ambient air (also in Tedlar bags).

The device operated in two different sampling modes based on the CO_2_-content. Digitally controlled electronic valves switched to sampling mode if (a) the absolute level of CO_2_ in the breath exceeded 3% or (b) the relative level of CO_2_ in the breath was above 80% of the maximal CO_2_-level in previous exhalation. Two breath samples and respective indoor-air were collected in described above way from each subject. Before use, all bags were thoroughly cleaned to remove any residual contaminants by flushing with nitrogen 6.0 (purity of 99.9999%), heating at 85°C (while filled with N_2_) for more than 8 hours and subsequent secondary flushing.

The study was approved by the local ethics committee of Innsbruck Medical University.

### Preparation of breath samples

Tedlar® bags filled with breath samples were thermostated for few minutes in an incubator at 40°C (to prevent condensation) and connected by means of Teflon tubes to a multibed sorption tube. The sample flow rate of 20 ml/min was diluted with additional flow (40 ml/min) of dry nitrogen 6.0 (additionally purified with Carboxen 1000) in order to avoid excessive adsorption of water vapor. The volume of the breath sample was 500 ml with a total flow of 60 ml/min through the multibed sorption tube, controlled by means of mass flow regulator (RED-Y, Burde Co. GmbH, Austria). For generation of sample flow a membrane pump (Vacuubrand, Wertheim, Germany) was placed at the end of sampling system.

Additional information (e.g. composition of sorption tubes, thermal desorption GC-MS settings) is provided elsewhere [61-64].

### Statistical analysis

Statistical significance was calculated by the Kruskal-Wallis test, which is a non-parametric test to compare samples from two or more groups of independent observations [65]. P-values <0.05 were considered to be significant. This test was selected because it does not require the groups to be normally distributed and is more stable to outliers. To summarize the data, results are plotted as median values with 5, 25, 75 and 95 percentiles. CFU counts are presented as mean values ± standard deviation (SD).

## Abbreviations

VAP, Ventilator associated pneumonia; ICU, Intensive care unit; VOCs, Volatile organic compounds; VSCs, Volatile sulfur-containing compounds; VNCs, Volatile nitrogen-containing compounds; CFU, Colony forming units; OD, Optical density; GC-MS, Gas chromatography mass spectrometry; SIFT-MS, Selected ion flow tube mass spectrometry; PTR-MS, Proton transfer reaction mass spectrometry; EN, Electronic nose; 2-AA, 2-aminoacetophenon; CF, Cystic fibrosis; BAL, Bronchoalveolar lavage; pptv, Part per trillion per volume (corresponding to the ratio of 1:10-12); ppbv, Part per billion per volume (corresponding to the ratio of 1:10-9); ppmv, Part per million per volume (corresponding to the ratio of 1:10-6); TIC, Total ion chromatogram; SIM, Selected ion monitoring; MeSH, Methanethiol; DMS, Dimethyl sulfide; DMDS, Dimethyl disulfide; DMTS, Dimethyl trisulfide; SPME, Solid phase microextraction; LOD, Limit of detection; MEP, Methylerythritol phosphate; COPD, Chronic obstructive pulmonary disease; SD, Standard deviation.

## Competing interests

Authors report no competing interests.

## Authors’ contribution

WF has developed the protocol for TD-GC-MS analyses of volatile compounds in headspace of cell cultures, including: conditions of sample collection, thermal desorption, GC temperature program, and mass spectrometry settings (SIM mode). Additionally, WF performed the gas chromatographic analysis of all samples, performed the calibrations, and wrote a draft of the manuscript. AS has contributed to cell culture sampling system development, performed the cell culture experiments and wrote the draft of the manuscript. MB has performed in part the cell culture experiments. AF has acquisitioned, analyzed and interpreted the chromatographic data. CA did the statistical analysis. HW has designed and constructed the system for bacteria cultivation and collection of headspace samples. MN, JT and AA have designed the study, discussed the results and finalized the manuscript. All authors read and approved the final manuscript.
